# The Villain Who Saved the Day: The Paradoxical Value of Amiodarone in Managing Recurrent Ventricular Tachycardia Secondary to Amiodarone-Induced Thyroiditis

**DOI:** 10.7759/cureus.55082

**Published:** 2024-02-27

**Authors:** Rakan M Alotaibi, Khaled M Abdullah, Cheng Ken Ong, Vetton Chee Kay Lee, Jing W Goh

**Affiliations:** 1 Internal Medicine, McGill University, Montréal, CAN; 2 Internal Medicine, King Faisal Specialist Hospital and Research Center, Madinah, SAU; 3 Internal Medicine, University of California, San Francisco, Fresno, USA; 4 Cardiology, Northern Care Alliance NHS Foundation Trust, Manchester, GBR; 5 Cardiology, Royal Perth Hospital, Perth, AUS; 6 Respiratory Medicine, Heartlands Hospital, Birmingham, GBR

**Keywords:** arrhythmia, ventricular arrhythmia, cardiology, tachy-arrhythmia, amiodarone, thyroiditis, amiodarone-induced thyroiditis, amiodarone-induced hyperthyroidism, ventricular tachycardia

## Abstract

Thyroiditis is a rare and serious complication for patients taking amiodarone. It can manifest with symptoms of hyperthyroidism and serious life-threatening arrhythmias. We present a case of a patient with amiodarone-induced thyrotoxicosis presenting with an electrical storm in which rhythm control was achieved with the utilization of amiodarone.

## Introduction

Amiodarone, a type III antiarrhythmic, is approved for the treatment of ventricular and supraventricular tachyarrhythmias [1]. Side effects are numerous, including both hypothyroidism and hyperthyroidism [2]. Although both are possible complications of amiodarone, the primary focus of this discussion will revolve around hyperthyroidism, particularly relating to arrhythmogenesis with overt thyroid dysfunction and the value of considering amiodarone in patients presenting with tachyarrhythmias associated with amiodarone-induced thyrotoxicosis (AIT). Approximately 14%-18% of patients on amiodarone may experience both thyrotoxicosis and hypothyroidism [3]. AIT is seen more commonly in regions with an iodine deficiency [4]. Conversely, amiodarone-induced hypothyroidism is seen in areas with sufficient iodine intake [5].

AIT can result either from increased synthesis of thyroid hormones (type I) or excess hormone release due to destructive thyroiditis (type II) [6]. In practice, both subtypes may clinically overlap, posing a considerable challenge for physicians to differentiate. Thus, management may include a combination of prednisone and antithyroid therapy [6,7]. Although mild AIT may subside spontaneously in 20% of patients [8], early recognition and management are critical as these patients were found to have a high incidence of mortality and morbidity [9].

## Case presentation

A 46-year-old gentleman presented to the emergency department due to four intracardiac defibrillator (ICD) shocks within the last 24 hours while conscious and asymptomatic. On further questioning, he reported experiencing hot flashes for the past two months and has been on amiodarone since 2018 for recurrent ventricular arrhythmias. Vital signs were significant for tachycardia ranging from 140 to 150 beats per minute. On physical examination, he was alert, distressed, and mildly breathless with no lid lag or goiter. He had pansystolic murmurs at the apex with an irregularly irregular rhythm with no bilateral pedal edema. His lungs were clear to auscultation bilaterally. ICD interrogation confirmed episodes of atrial fibrillation and non-sustained ventricular tachycardia (VT), for which appropriate shocks were delivered. He had regular three-monthly cardiology outpatient follow-up with ICD analysis for arrhythmias which were normal before this admission.

The patient has a past medical history of chronic congestive heart failure (New York Heart Association class IV, American College of Cardiology stage D) secondary to Becker Muscular Dystrophy. His latest echocardiogram performed one month before this admission showed severely impaired left ventricular function with an estimated left ventricular ejection fraction of 10%-15% as well as moderate to severe mitral regurgitation. He had a primary prevention subcutaneous ICD implanted in 2013. His regular medications include amiodarone, bisoprolol, rivaroxaban, furosemide, ramipril, and ivabradine.

Given this patient’s history of severe congestive heart failure, the risk of VT significantly increases especially in the setting of other possible triggers. The possible etiologies of ventricular arrhythmia in this patient likely include anemia, dehydration, hyperthyroidism, electrolyte abnormalities, or toxins. Other causes may also include ischemia, pulmonary embolism, or anxiety.

**Figure 1:**
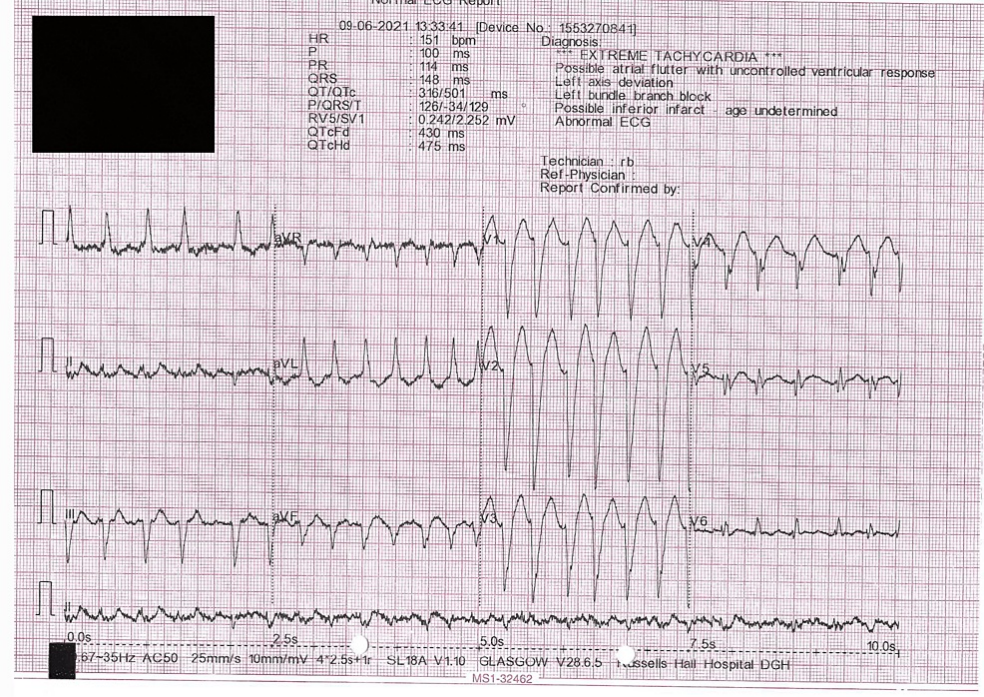
EKG on Presentation EKG: Electrocardiogram.

On admission, EKG (Figure 1) showed regular broad complex VT with a heart rate of 151 beats per minute and a wide QRS complex of 148ms. Table 1 displays the blood results on admission and four months ago. Endocrinology was subsequently consulted in view of a deranged thyroid function test on admission suggesting hyperthyroidism. Further workup revealed that both anti-TSH receptor antibodies and anti-thyroid peroxidase antibodies (TPO) were negative. The thyroid isotope scan conducted showed no uptake of the tracer within the thyroid gland. Thus, the findings were more consistent with type 2 AIT.

**Table 1:** Laboratory tests Table Credits: Authors' own creation. TSH: Thyroid-Stimulating Hormone; FT4: Free Thyroxine 4; FT3: Free Triiodothyronine 3; WBC: White Blood Cell; GFR: Glomerular Filtration Rate; CRP: C-Reactive Protein.

Test (unit)	Results (four months ago)	Results (Upon Admission)	Reference Range
TSH (mU/L)	0.68	< 0.01↓	0.4-4
FT4 (pmol/L)	-	53.1 ↑	10-28
FT3 (pmol/L)	-	6.8	4.6-9.7
WBC Count (K/uL)	8.4	9.0	4.5-11
Hemoglobin (g/L)	140	156	Male: 138-172 Female: 121-151
Platelets (x10^9/L)	319	276	150-450
Sodium (mmol/L)	139	140	136-145
Potassium (mmol/L)	4.6	4.8	3.6-5.2
GFR (mL/min)	>90	>90	>60
CRP (mg/L)	-	8.0	0-5
Troponin (ng/L)	-	22.0	0-14
Magnesium (mmol/L)	0.85	0.9	0.65-1.05

**Figure 2:**
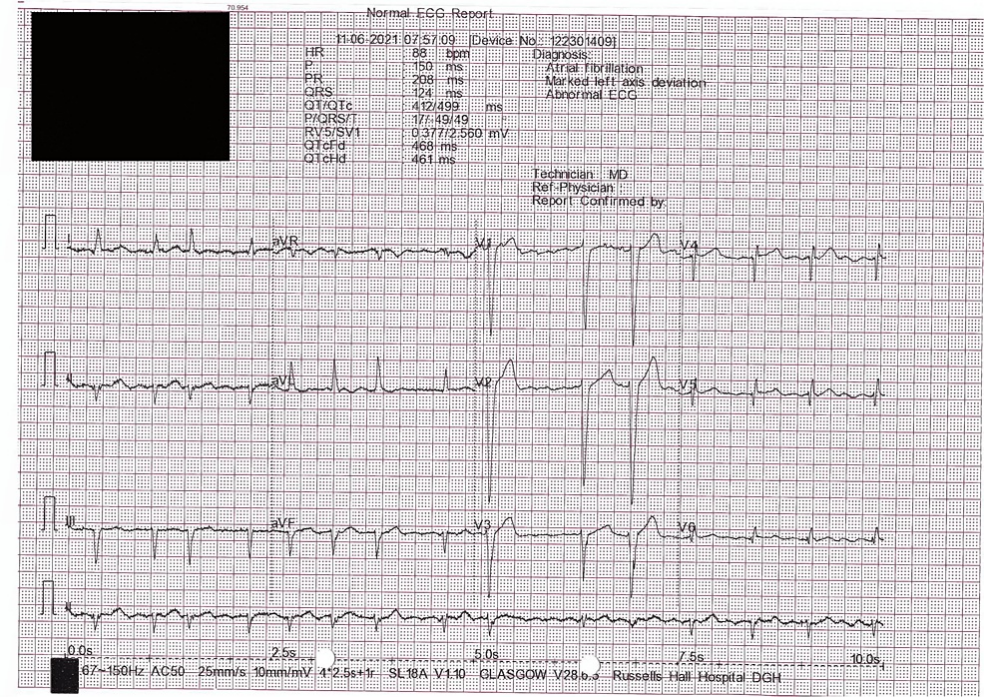
EKG prior discharge EKG: Electrocardiogram

The patient was started on intravenous magnesium, propylthiouracil and prednisolone as advised by the endocrinology team. He was started on intravenous amiodarone (150 mg administered intravenously over 10 minutes, followed by continuous intravenous infusion rate of 1 mg/min for six hours, then at an infusion rate of 0.5 mg/min for an additional 18 hours). His Amiodarone therapy of 200 mg orally once daily was then continued to control the risk of life-threatening ventricular arrhythmias. The patient reverted to rate-controlled atrial fibrillation (Figure 2) from day 3 of treatment and had a successful discharge home. His Amiodarone home dose is not changed after discharge.

The patient was advised to follow up with the cardiology and endocrinology clinics after hospital discharge. Following discussion and shared decision making with the patient, the endocrinologist has recommended the patient to be on propylthiouracil and prednisolone for AIT. The cardiologist advised the continuation of amiodarone as it was the most effective in achieving rhythm control for this patient compared to other antiarrhythmic agents.

Following three months from his previous discharge, the patient reported no symptoms of thyrotoxicosis and his thyroid levels had normalized. Thus, prednisolone was tapered and subsequently discontinued six months after his hospital discharge. He continued to be on propylthiouracil as he was not deemed suitable for thyroidectomy due to his severe heart failure.

## Discussion

We report a 46-year-old gentleman who presented with ICD shocks while being hemodynamically stable, with a known medical history of Becker Muscular Dystrophy who has been on amiodarone, workup was consistent with AIT type 2. Studies have generally outlined two subtypes of AIT which are summarized in Table 2 [6].

**Table 2:** Type 1 vs type 2 AIT. AIT: Amiodarone-Induced-Thyrotoxicosis; T4: Thyroxine; T3: triiodothyronine; CFDS: Color-Flow Doppler Ultrasonography [4].

Features	Type 1 AIT	Type 2 AIT
↑ T4 and T3 Mechanism	Excessive synthesis	Excessive release (destructive thyroiditis)
Goiter	Often multinodular or diffuse	Often absent or small
Underlying Thyroid Disease	Usually latent Grave’s Disease	Usually absent
Thyroglobulin	↑	↓ (relatively)
24-hour Radioiodine Uptake	Detectable	No uptake
CFDS	↑ vascularity	↓ vascularity
Management	Thionamides + Surgery	Glucocorticoids + Surgery

Ventricular arrhythmias are serious and a potentially fatal consequence of AIT. In a retrospective study, the incidence of AIT was associated with a 2.7-fold increased risk of adverse cardiovascular events including myocardial infarction, heart failure, and ventricular arrhythmias when compared to euthyroid patients on amiodarone [10]. Moreover, an electrical storm is possible in patients with thyrotoxicosis and is defined, in patients with ICD, as three appropriate VT detections in 24 hours, treated by pacing, shock, or eventually untreated but closely monitored [11]. In our case, the patient sustained four ICD shocks within the last 24 hours and thus meets the definition of an electrical storm. Because the initial cause of thyrotoxicosis and arrhythmogenesis can be linked to amiodarone, physicians may consider an alternative management option to amiodarone for rhythm control when suspecting AIT.

In our case, the patient sustained four ICD shocks within the last 24 hours and thus meets the definition of an electrical storm. Because the initial cause of thyrotoxicosis and arrhythmogenesis can be linked to amiodarone, physicians may consider an alternative management option to amiodarone for rhythm control when suspecting AIT. However, in our case, it was deemed to continue Amiodarone in the setting of AIT given it is the first-line treatment for VT and continuing amiodarone is beneficial in such a case as the patient was receiving ICD shocks. Furthermore, Amiodarone remains the only guideline-recommended therapy for secondary VT with either ischemic or non-ischemic cardiomyopathy [12]. The use of other antiarrhythmic such as flecainide and propafenone are contraindicated in patients with structural heart disease including left ventricular dysfunction as it was associated with ventricular proarrhythmia [13,14].

In the immediate setting, stopping the drug is unlikely to have a major impact as the drug has an elimination half-life of approximately 100 days [15]. In hemodynamically unstable patients with VT, electrical cardioversion is recommended. In stable patients with VT, it is recommended to initially manage the patient with IV amiodarone and an oral beta-blocker [12]. In our case, amiodarone was able to achieve rhythm control. However, the management’s complexity increases considerably when considering long-term treatment. One study reported on a 56 male who had been on amiodarone for atrial fibrillation and presented with type 2 AIT. The patient was managed with discontinuing amiodarone and starting propylthiouracil; however, on outpatient follow-up the thyroid function test was still in the thyrotoxic range, hence requiring adding oral prednisolone with eventual resolution [16]. Additionally, Inaba et al reported a 61-year-old male who presented with thyrotoxicosis symptoms and had been on amiodarone for VT. Eventually workup showed AIT type I with papillary thyroid cancer and multinodular goiter. Amiodarone was discontinued and methimazole was started with a reported improved thyroid function test after 5 weeks and the patient was considered for total thyroidectomy [17].

Moreover, a retrospective cohort study has demonstrated that in circumstances where patients do not respond to medical treatment, require immediate improvement, or necessitate continued amiodarone treatment, a total thyroidectomy under general anesthesia is an effective and proper treatment option [18].

Although careful clinical judgment and decision are crucial for the appropriate management of such patients, we hope this discussion will encourage further reports and studies to gather more evidence to implement standardized guidelines on the use of amiodarone in this challenging subset of patients with AIT.

## Conclusions

A thorough history and physical examination can help identify patients suffering from AIT. Differentiation between the subtypes can be challenging, therefore, management options tend to overlap the two subtypes. Long-term treatment of AIT is complex due to factors further complicating treatment decisions such as considering the importance of amiodarone in the management of arrhythmias. Despite relating to the initial pathogenesis, amiodarone may have a considerable role in achieving rhythm control in such patients.
